# Genetic Analysis of *Bordetella pertussis* Isolates from the 2008–2010 Pertussis Epidemic in Japan

**DOI:** 10.1371/journal.pone.0077165

**Published:** 2013-10-04

**Authors:** Yusuke Miyaji, Nao Otsuka, Hiromi Toyoizumi-Ajisaka, Keigo Shibayama, Kazunari Kamachi

**Affiliations:** 1 Department of Bacteriology II, National Institute of Infectious Diseases, Tokyo, Japan; 2 Department of Pediatrics, St Marianna University School of Medicine, Kawasaki, Japan; University of Edinburgh, United Kingdom

## Abstract

A large pertussis epidemic occurred between 2008 and 2010 in Japan. To investigate epidemic strains, we analyzed 33 *Bordetella pertussis* isolates from the epidemic period by sequencing virulence-associated genes (*fim3*, *ptxP*, *ptxA*, and *prn*) and performing multilocus variable-number tandem repeat analysis (MLVA), and compared these results with those of 101 isolates from non-epidemic, earlier and later time periods. DNA sequencing of the *fim3* allele revealed that the frequency of *fim3B* was 4.3%, 12.8%, 30.3%, and 5.1% within isolates in 2002–2004, 2005–2007, 2008–2010, and 2011–2012, respectively. The isolation rate of the *fim3B* strain therefore temporarily increased during the epidemic period 2008–2010. In contrast, the frequencies of the virulence-associated allelic variants, *ptxP3*, *ptxA1*, and *prn2*, increased with time during overall study period, indicating that these variants were not directly involved in the occurrence of the 2008–2010 epidemic. MLVA genotyping in combination with analysis of allele types showed that the prevalence of an MT27d strain temporarily increased in the epidemic period, and that this strain carried virulence-associated allelic variants (*fim3B*, *ptxP3*, *ptxA1*, and *prn2*) also identified in recent epidemic strains of Australia, Europe, and the US. Phenotypic analyses revealed that the serotype Fim3 strain was predominant (≥87%) during all the periods studied, and that the frequency of adhesion pertactin (Prn) non-expressing *B. pertussis* decreased by half in the epidemic period. All MT27d strains expressed Prn and Fim3 proteins, suggesting that *B. pertussis* MT27d strains expressing Prn and Fim3B have the potential to cause large epidemics worldwide.

## Introduction

*Bordetella pertussis*, a highly communicable Gram-negative coccobacillus, is the cause of pertussis (whooping cough), a major acute respiratory infection resulting in severe childhood illness and infant death [1]. Although universal immunization programs have contributed to significant reductions in the morbidity and mortality rates associated with pertussis, the incidence of pertussis has increased in several countries despite high vaccination coverage [2]-[6]. In Japan, acellular pertussis vaccines (ACVs) were introduced in 1981 and are used to control pertussis with a schedule of 3 primary doses and single booster dose at ages 3, 4, 6, and 18−23 months, respectively. This vaccination schedule has been followed since 1994. The incidence of pertussis cases in adolescents and adults, however, has significantly increased since the early 2000s [7]. The waning of vaccine-acquired immunity and the decrease in opportunities of natural immune boosting owing to reduced levels of *B. pertussis* circulation have been proposed as explanations for the recent resurgences of pertussis [2], [8], [9]. Another possible underlying factor is the adaptation of the *B. pertussis* population to vaccine-induced immunity [2], [10], [11].

Antigenic and genetic shifts in *B. pertussis* circulating strains have been identified within virulence-associated genes encoding serotype 3 fimbriae (*fim3*), pertussis toxin S1 subunit (*ptxA*), pertactin (*prn*), and the pertussis toxin promoter (*ptxP*). Allele frequencies of the virulence-associated allelic variants, *fim3B*, *ptxA1*, *prn2,* and *ptxP3*, have increased within the *B. pertussis* population in several countries [10], [12]–[17]. *B. pertussis* strains carrying *ptxP3* are more capable of producing pertussis toxin (PT) than are *ptxP1* strains, and the emergence of *ptxP3* strains was associated with pertussis resurgence in the Netherlands [18]. Similarly, a significant correlation was observed between an increase in *fim3B* strains and pertussis notifications in the US [13]. Strains with *fim3B* have a single amino-acid substitution (A87E) within the surface epitope of Fim3, which interacts with human serum [19], [20]. Furthermore, multilocus variable-number tandem repeat analysis (MLVA) has revealed that the *B. pertussis* population has changed over the past 50 years worldwide. In Australia, the frequency of *B. pertussis* MLVA type 27 (MT27) and MT70 strains increased after the introduction of an ACV, and subsequently, the MT27 strain became predominant in 2008−2010 [21], [22]. An increase in the frequency of *B. pertussis* MT27 strain was also observed in Europe and the US [13], [16], [23].

Pertussis epidemics still occur worldwide, and epidemic strains have been characterized by Fim serotyping and/or genotyping within some regions [24]–[26]. In a Dutch epidemic, significant changes in Fim serotypes and MTs were observed during a period when the pertussis vaccine dose was lowered [24]. Besides phenotypic variants of Fim, *B. pertussis* variants that do not express adhesion pertactin (Prn) have been recently identified in Japan as well as in other countries [27]–[30]. Since Prn is a component of ACVs, it is reasonable to hypothesize that Prn-negative variants have increased fitness in humans immunized with ACV. To date, the relationship between the prevalence of Prn-negative variants and pertussis epidemics has not been evaluated.

In Japan, a large pertussis epidemic occurred in 2008−2010 despite high vaccination coverage with ACVs. To elucidate the causes of the epidemic, we determined temporal trends in the frequencies of virulence-associated genes (*fim3*, *ptxP*, *ptxA*, and *prn*) and genotypes in the *B. pertussis* population from 2002 to 2012. In addition, phenotypes of epidemic isolates were characterized by their expression of Fim and Prn proteins.

## Materials and Methods

### Pertussis surveillance data

National surveillance data were obtained from the Ministry of Health, Labor and Welfare of Japan Infectious Disease Surveillance data. Each week, the number of pertussis cases was reported from approximately 3,000 sentinel clinics and hospitals within Japan. Diagnosis was based on bacterial culture, clinical symptoms, and/or the results of a serologic test. The reporting criteria did not change during the study period, 2002−2012.

### Bacterial strains

**Figure 1:**
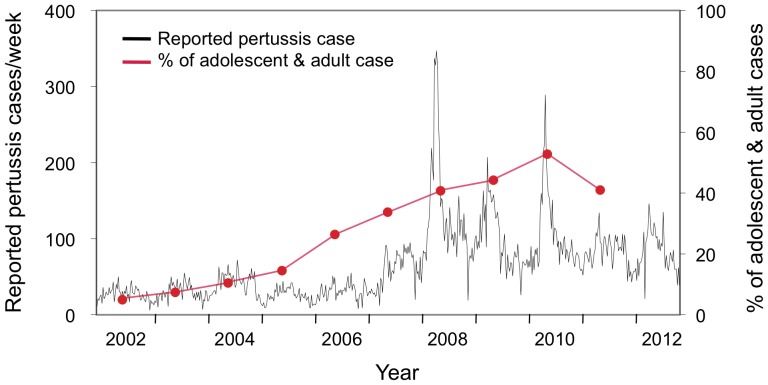
Number of reported pertussis cases per week in Japan from 2002 to 2012. Pertussis cases are shown by the black line, with each value representing a week of the year. The percentage of adolescent and adult cases (≥15 years old) per year is shown in red circles. The data were obtained from the Ministry of Health, Labor and Welfare of Japan Infectious Disease Surveillance data. Data regarding the number of adolescent and adult cases in 2012 were not available.

We examined 134 clinical *B. pertussis* isolates collected in Japan from 2002 to 2012 (Table S1). Thirty-three of those isolates were collected during the 2008–2010 pertussis epidemic, while 101 isolates were collected from non-epidemic periods: 23 in 2002–2004, 39 in 2005–2007, and 39 in 2011–2012 (Fig. 1). All isolates were epidemiologically unrelated cases of pertussis. The isolates were cultured on Bordet-Gengou agar (Difco) or cyclodextrin solid medium (CSM) agar [31], and incubated at 36°C for 2−3 days. DNA was extracted from *B. pertussis* isolates by boiling, and stored at −20°C.

### Sequence analysis of *fim3*, *ptxP*, *ptxA*, and *prn*

Four virulence-associated genes, *fim3*, *ptxP*, *ptxA* and *prn*, were analyzed using PCR-based sequencing [14], [18], [32], [33]. Sequence reactions were carried out with a BigDye® Terminator v3.1 Cycle Sequencing Kit (Applied Biosystems), and resultant products were sequenced using an Applied Biosystems 3130xl Genetic Analyzer or 3730 DNA Analyzer. Subsequent sequencing of the variable region 2 (R2) of *prn* was performed where necessary, to distinguish between *prn1* and *prn7* alleles. Primer sets used in this study are listed in Table S2.

### MLVA

MLVA typing was performed as previously described [22], [28]. MTs were assigned using the MLVA typing tool found at http://www.mlva.net. Novel MLVA types were assigned by the webmasters, Drs. H. van der Heide and F. Mooi, from the Centre for Infectious Disease Control Netherlands, within National Institute for Public Health and the Environment, in the Netherlands.

### Immunoblotting and serotyping analysis

Prn expression in *B. pertussis* isolates was analyzed by immunoblotting [28]. Briefly, protein samples (1 µg) were first subjected to 10% SDS-PAGE, then transferred onto nitrocellulose membranes (Bio-Rad), and finally incubated with anti-Prn antiserum. Antigen-antibody complexes were visualized using a horseradish peroxidase-conjugated secondary antibody (Bio-Rad) and the Western Lightning ECL Pro (PerkinElmer, Inc.). Resultant blots were imaged using a LAS-3000 (Fujifilm, Tokyo, Japan).

Serotyping of *B. pertussis* isolates was performed with indirect whole-cell ELISA using anti-Fim2 and anti-Fim3 monoclonal antibodies as previously described [34], [35], with some minor modifications. Briefly, bacterial cells cultured on Bordet-Gengou agar were resuspended in phosphate-buffered saline (PBS) to an optical density of 0.01 at 620 nm, and then inactivated at 56°C for 1 h. The wells of 96-well ELISA plate (Nunc Immuno Plate Maxisorp) were coated with 100 µl of this suspension to each well and allowing it to evaporate overnight at 36°C. Anti-Fim2 (NIBSC 04/154) and anti-Fim3 (NIBSC 04/156) antibodies were used at a 1∶1,000 dilution in PBS. Antibody binding to bacterial cells was detected following the addition of a 1∶4,000 dilution of alkaline phosphatase-labeled goat anti-mouse IgG (Southern Biotechnology Associates, Inc.) and with the use of *p*-nitrophenylphosphate as a substrate. The optical density was measured at 405 nm with 650 nm as a reference using a Multiskan FC microplate reader (Thermo Fisher Scientific Inc.). *B. pertussis* strain 18323 that expresses both Fim2 and Fim3 was used as a positive control.

### Statistical analysis

Squared Pearson’s correlation coefficient (R^2^) was used to identify a linear dependence between allele frequency (*fim3B*, *ptxP3*, *ptxA1,* or *prn2*) and isolation periods. Fisher’s exact test was performed to analyze the distribution of *B. pertussis* population. The Simpson’s diversity index (DI) and 95% confidence interval (CI) of MTs was calculated as described by Hunter and Gaston [36] and Grundmann et al. [37], respectively, using the online tool available at http://www.comparingpartitions.info/.

## Results

### Characteristics of the 2008−2010 pertussis epidemic in Japan

There were 17,349 reported pertussis cases between January 2008 and December 2010 in Japan. Within this pertussis epidemic period, 3 sharp peaks representing increases in case frequency were observed: 1 in late May 2008 (347 cases at week 22), 1 in mid-May 2009 (207 cases at week 20), and 1 in mid-June 2010 (289 cases at week 24) (Fig. 1). The number of reported cases per year in 2008−2010 was ≥2.7 times higher than the previous 5-year average. Although the number of pertussis patients over 15 years of age steadily increased in the 2000s, alongside increases of adolescent and adult incidence rates (40.7%, 44.2%, and 52.9% of all reported cases in 2008, 2009, and 2010, respectively), in 2011, the number of those patients decreased and the incidence rate in adolescents and adults also decreased to 41%.

### Temporal changes in the frequencies of *fim3*, *ptxP*, *ptxA,* and *prn* alleles

**Figure 2:**
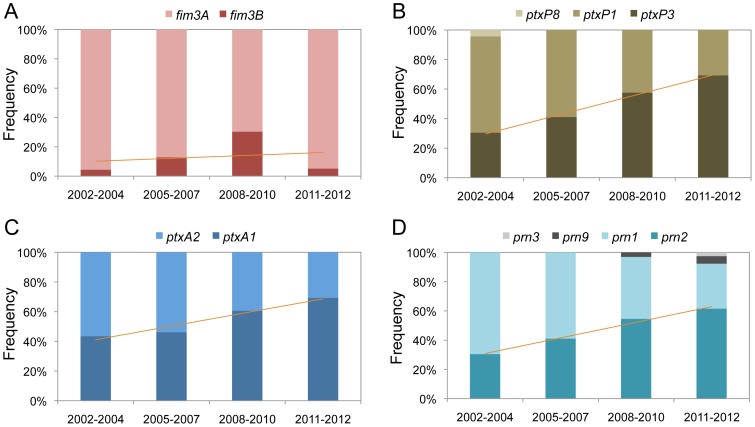
Temporal trends in the frequencies of *fim3*, *ptxP*, *ptxA*, and *prn* alleles within *Bordetella pertussis* isolates in Japan from 2002 to 2012. Four allelic genes, *fim3* (A), *ptxP* (B), *ptxA* (C), and *prn* (D), of 134 *B. pertussis* isolates were sequenced. Isolate allele frequencies are shown by time period: 2002−2004 (non-epidemic, n = 23), 2005−2007 (pre-epidemic, n = 39), 2008−2010 (epidemic, n = 33), and 2011−2012 (post-epidemic, n = 39). The regression line shows the relationship between the frequency of virulence-associated allelic variant (*fim3B*, *ptxP3*, *ptxA1,* or *prn2*) and the 4 time periods.

Among the 134 *B. pertussis* isolates tested, 2 *fim3* (*fim3A* and *fim3B*), 3 *ptxP* (*ptxP1*, *ptxP3* and *ptxP8*), 2 *ptxA* (*ptxA1* and *ptxA2*), and 4 *prn* (*prn1*, *prn2*, *prn3,* and *prn9*) alleles were identified. Figure 2 shows the temporal trends of the allele frequencies. The frequency of the allele *fim3B* temporarily increased during the epidemic period (2008–2010): it was 4.3% in 2002–2004, 12.8% in 2005–2007, 30.3% in 2008–2010, and 5.1% in 2011–2012 (Fig. 2A). In contrast, the frequencies of *ptxP3*, *ptxA1*, and *prn2* increased with time from 2002 to 2012 (Fig. 2B-D). High correlations (R^2^ ≥ 0.95) were observed between these latter allele frequencies and the isolation periods.

### Temporal changes in the frequencies of Prn and Fim variants

**Figure 3:**
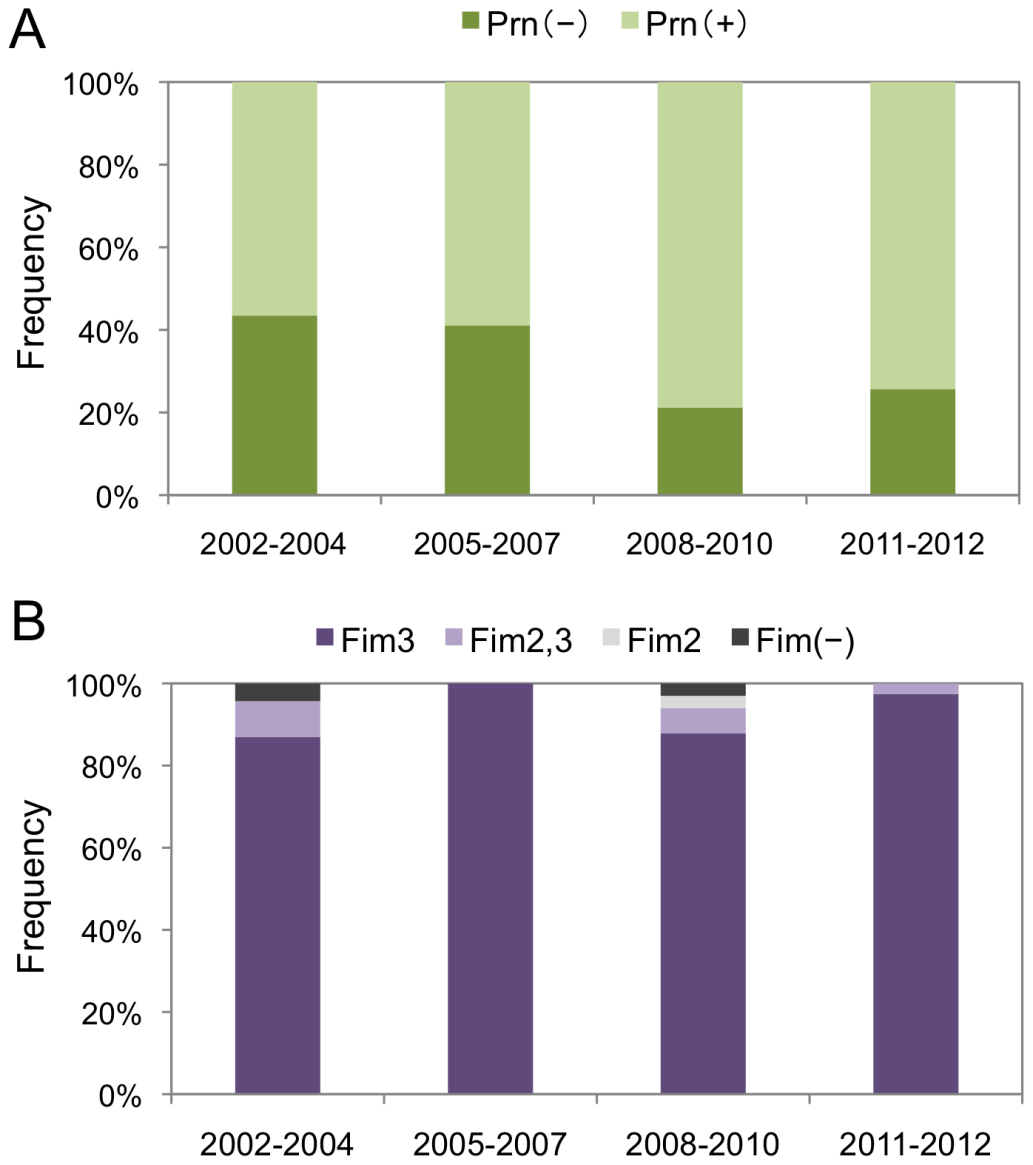
Temporal trends in the frequencies of Prn and Fim3 variants of *Bordetella pertussis* isolates in Japan from 2002 to 2012. Prn (A) and Fim (B) expression was analyzed within 134 *B. pertussis* isolates. Two Prn variants, Prn(+) and Prn(−), and 4 Fim variants, Fim2, Fim3, Fim2,3, and Fim(−) strains, were identified. Variant frequencies are shown by time period: 2002−2004 (non-epidemic, n = 23), 2005−2007 (pre-epidemic, n = 39), 2008−2010 (epidemic, n = 33), and 2011−2012 (post-epidemic, n = 39).

The Prn and Fim expression phenotypes of the 134 *B. pertussis* study isolates were determined with immunoblotting and indirect whole-cell ELISA, respectively. Figure 3A shows the temporal trend of the frequencies of the 2 identified Prn variants, Prn-expressing and Prn-negative strains, with the frequency of Prn-negative strains at 43.5%, 41.0%, 21.2%, and 25.6% during 2002–2004, 2005–2007, 2008–2010, and 2011–2012, respectively. A decreased frequency of Prn-negative strains was observed during the epidemic period (2008–2010); however, this decrease was not statistically significant (*P*>0.05). All the Prn-negative strains carried the *prn1* allele (Table S1). Prn-negative strains carrying *prn1* were previously found in Finland [30], and those carrying *prn2* were found in France and the US [27], [29].

On the other hand, 4 Fim variants, Fim2, Fim3, Fim2,3, and Fim(−), were identified among the *B. pertussis* isolates. The Fim2,3 strain was detected by both Fim2 and Fim3 antigens, while the Fim(−) strain was not detected by either. As shown in Figure 3B, the Fim3 strain was predominant during all the time periods studied: 87.0% in 2002−2004, 100% in 2005−2007, 87.9% in 2008−2010, and 97.4% in 2011−2012. Two Fim(−) strains were isolated in 2002−2004 and 2008−2010. Interestingly, one Fim(−) strain was previously identified in Ontario, Canada [12].

### Temporal changes in MTs and genetic diversity

**Figure 4:**
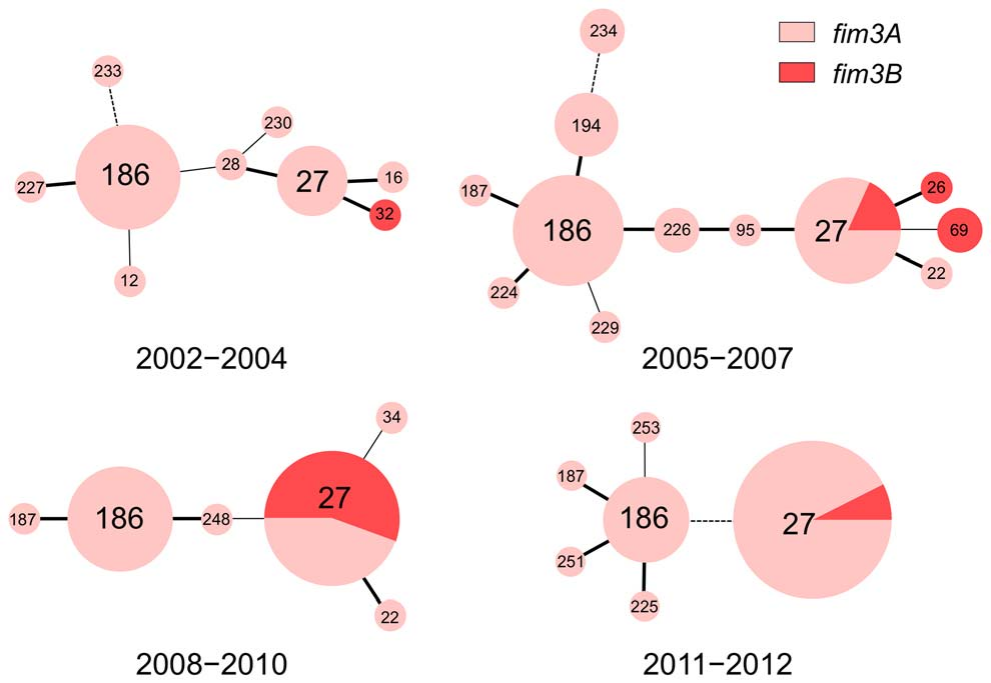
Minimum spanning trees revealing the genetic diversity of the *Bordetella pertussis* population in Japan from 2002 to 2012. MTs were determined for 134 *B. pertussis* isolates. The resultant phylogenetic trees based on MTs are shown by time period: 2002−2004 (non-epidemic, n = 23), 2005−2007 (pre-epidemic, n = 39), 2008−2010 (epidemic, n = 33), and 2011−2012 (post-epidemic, n = 39). Each circle within a tree represents a different MT, with the MT number noted. Thick lines separate single-locus variants, while thin lines separate double-locus variants, and dotted lines signify a more distant relationship. Pink and red colors indicate, respectively, the *fim3A* and *fim3B* allele frequencies within MTs.

Among the 134 *B. pertussis* study isolates, 24 different MTs were identified, of which 2 were novel (MT251 and MT253). Figure 4 shows minimum spanning trees that revealed the genetic diversity of the *B. pertussis* population during the time periods of 2002−2004, 2005−2007, 2008−2010, and 2011−2012. Eighteen *B. pertussis* isolates carrying *fim3B* were identified during the 4 time periods, and these *fim3B* strains were divided into 4 MTs: MT26 (n = 1), MT27 (n = 14), MT32 (n = 1), and MT69 (n = 2). Although MT27 and MT186 were the predominant types during all the time periods, the *fim3B* strain did not belong to MT186. MT27 strains carrying *fim3B* were most frequent in MT27 during the epidemic period (2008−2010) at 56% (10/18), and their frequency decreased to 7% (2/27) in 2011−2012. The temporal increase of MT27 strains carrying *fim3B* was statistically significant (*P* < 0.05) when compared with non-MT27 strains carrying *fim3B*.

MT27 strains could be further classified into 5 subtypes (MT27a to MT27e) based on their allele types (Table 1). MT27a strains carried *fim3A, ptxP3*, *ptxA1*, and *prn2*, and were the predominant subtype. Notably, they were collected in both non-epidemic and epidemic periods. In contrast, MT27d strains carrying *fim3B*, *ptxP3*, *ptxA1*, and *prn2* were predominantly isolated during the epidemic period, with 10 of 12 isolates (83%) being of this subtype in 2008−2010. An MT27c strain carrying *fim3A*, *ptxP3*, *ptxA1*, and *prn9* was also isolated during the epidemic period. The other MT27 subtypes, MT27b and MT27e, were found only in 2011−2012.

**Table 1:** Comparison of MTs and allele types between *Bordetella pertussis* isolates collected in non-epidemic and epidemic periods.

	Allele types				No. of isolates detected within the time period			
MT	*fim3*	*ptxP*	*ptxA*	*prn*	2002–2004	2005–2007	2008–2010*	2010–2012
12	A	8	2	1	1			
16	A	3	1	2	1			
22	A	3	1	2		1	1	
26	B	3	1	2		1		
27a	A	3	1	2	5	9	7	24
27b	A	3	1	3				1
27c	A	3	1	9			1	
27d	B	3	1	2		2	10	
27e	B	3	1	9				2
28	A	1	1	1	1			
32	B	3	1	2	1			
34	A	1	1	1			1	
69	B	3	1	2		2		
95	A	3	1	2		1		
186	A	1	2	1	11	12	11	8
187	A	1	2	1		1	1	1
194	A	1	2	1		4		
224	A	1	2	1		1		
225	A	1	2	1				1
226	A	1	2	1		2		
227	A	1	2	1	1			
229	A	1	2	1		1		
230	A	1	1	1	1			
233	A	1	1	1	1			
234	A	1	1	1		2		
248	A	1	2	1			1	
251	A	1	2	1				1
253	A	1	2	1				1

* Pertussis epidemic period.

**Figure 5:**
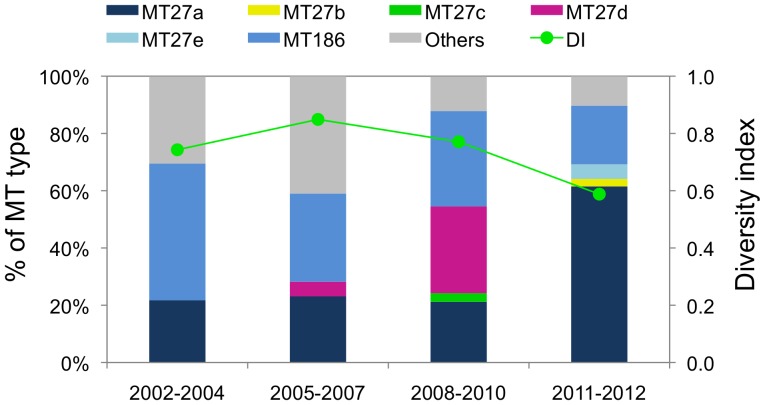
Frequency of MLVA types and genetic diversity of the *Bordetella pertussis* population in Japan from 2002 to 2012. The diversity index (DI) and 95% confidence interval (CI) were calculated from the MT frequencies within 4 time periods: 2002−2004 (non-epidemic), 2005−2007 (pre-epidemic), 2008−2010 (epidemic), and 2011−2012 (post-epidemic). For convenience, minor MTs (MT22, MT34, MT187, and MT248) are shown as “Others”.

The genetic diversity of MTs and the 5 MT27 subtypes was subsequently determined. As shown in Figure 5, Simpson’s DI was 0.74 (95% CI, 0.58−0.90), 0.85 (0.78−0.92), 0.77 (0.70−0.84), and 0.59 (0.43−0.75) in 2002−2004, 2005−2007, 2008−2010, and 2011−2012, respectively. Therefore, the genetic diversity within *B. pertussis* population decreased after the 2008−2010 pertussis epidemic.

### Relationship between MTs and phenotypes in 2008−2010

During the 2008−2010 pertussis epidemic, MT186 (33%), MT27a (21%), and MT27d (30%) were the predominant MTs, and most of them were serotype Fim3 strains (89%) (Table 2). Fim3 strains were also identified within other minor MTs (MT22, MT27c, MT187, and MT248), while a Fim2 strain belonged to MT34, the 2 Fim2,3 strains were typed as MT27d and MT186, and the Fim(−) strain belonged to MT27a. Of the 10 MT27d strains, 9 expressed Fim3 and 1 expressed both Fim2 and Fim3. On the other hand, Prn-negative strains were all observed within MT186. Seven (64%) of the 11 MT186 strains did not express Prn. All other MTs expressed Prn.

**Table 2:** Relationship between MTs and phenotypes in *Bordetella pertussis* isolates collected during the 2008−2010 pertussis epidemic period.

		Fim expression				Prn expression	
MT	No. of isolates	Fim2	Fim3	Fim2,3	Fim(−)	Prn(+)	Prn(−)
22	1		1			1	
27a	7		6		1	7	
27c	1		1			1	
27d	10		9	1		10	
34	1	1				1	
186	11		10	1		4	7
187	1		1			1	
248	1		1			1	

## Discussion

In the present study, we demonstrated that the prevalence of *B. pertussis* strains carrying *fim3B*, which were classified as MT27d, temporarily increased during the 2008–2010 pertussis epidemic in Japan. The MT27d strains of the epidemic period were collected in several areas (Table S1). All MT27d strains carried *fim3B*, *ptxP3*, *ptxA1*, and *prn2*, and expressed Prn and Fim3 proteins. Although Prn-negative strains have recently increased in their prevalence within Japan and in other countries [27]–[30], here, a lowered frequency of Prn-negative strains was observed during the epidemic period specifically, indicating that Prn-negative strain types were not involved in the epidemic. Similarly, we found that the prevalence of the virulence-associated allelic variants, *ptxP3*, *ptxA1*, and *prn2*, has increased with time from the early 2000s, indicating that the variants were also not directly associated with the epidemic. Taken together, our findings demonstrate that *B. pertussis* strains carrying *fim3B* (i.e., MT27d) were associated with the 2008–2010 pertussis epidemic.

We evaluated the regional effect on *B. pertussis* population because of the low number of samples of isolates. When the number of isolates was compared, the difference in regional population between epidemic (2008–2010) and post-epidemic (2011–2012) periods was statistically significant (*P* < 0.01), possibly because of the high number (18/39) of isolates collected in the Kinki district (including Osaka) during the post-epidemic period. A sampling bias cannot be excluded from the analysis of the trend in 2011–2012. However, no significant difference was observed between pre-epidemic (2005–2007) and epidemic periods (*P* > 0.05). The regional effect was therefore small or negligible for detection of the emergence of strain MT27d in the 2008–2010 epidemic.

In the past decade, the prevalence of *B. pertussis* strains carrying *fim3B* has increased worldwide [12]–[14], [25]. In Ontario, Canada, 1 predominant *B. pertussis* clone was identified in the 2000s [12]. This strain carried the same virulence-associated allelic variants (*fim3B*, *ptxP3*, *ptxA1* and *prn2*) as our epidemic strains within MT27d and was identical to the strains involved in recent pertussis resurgences within Europe and Australia. Similarly, *fim3B* strains carrying *ptxP3*, *ptxA1*, and *prn2* were predominant in the US during the 2000s, and most were genotyped to MT27 [13]. Interestingly, the pertussis resurgence in the US was correlated with the emergence and predominance of the *fim3B* allele, but not with the *ptxP3* allele. On the other hand, in the Netherlands, the prevalence of *fim3B* strains temporarily increased in the early 2000s, although the strains disappeared in 2010 [14]. Likewise, MT27d strains (carrying *fim3B*) were not identified after the 2008−2010 epidemic period, and the reasons behind the disappearance of this strain type are unclear. Our findings along with those of previous studies, therefore, suggest that the MT27d strain is a recent epidemic strain that is found worldwide, and that this strain is not only associated with pertussis resurgence but can also be correlated with pertussis epidemics.

Fimbriae of *B. pertussis* are composed of Fim2 and/or Fim3 and FimD. The minor fimbrial subunit FimD forms the adhesive tip [38]. Fim2 and Fim3 are encoded by the single-copy genes *fim2* and *fim3*, respectively [39], [40], and are serologically distinct [41]. Fim resulting from the *fim3B* strain is Fim3B, which differs from Fim3A by a single amino-acid substitution (A87E) at the surface epitope. To date, the biological differences between Fim3A and Fim3B are unknown. In an effort to address this issue, we recently observed *B. pertussis* clinical strains and found that most strains carrying *fim3B* had strong autoagglutination capability, unlike those carrying *fim3A*, following the suspension of CSM agar cultures into saline (Otsuka and Kamachi, unpublished data). Surprisingly, autoagglutination was not observed when the strains were cultured on Bordet-Gengou agar. Bacterial autoaggregation is a phenomenon associated with pathogen virulence in many Gram-negative bacteria [42]–[46], suggesting Fim3B strains are more virulent than Fim3A strains because of their ability to autoagglutinate. Further study is necessary to fully elucidate the relationship between autoagglutination and Fim3B. Attempts to identify the molecular mechanism that regulates autoagglutination within *fim3B* strains are currently underway.

In many countries, a shift from serotype Fim2 to Fim3 in *B. pertussis* circulating strains was observed after mass vaccination, and antigenic differences (in Fim, PT, and Prn) have been since noted between *B. pertussis* vaccine strains and circulating strains. In Japan, the *B. pertussis* strain Tohama carrying *ptxA2*, *prn1*, and *fim2* has been used as a vaccine strain to produce ACVs from 1981. Among the 4 currently used Japanese ACVs, 2 contain Fim2 and all do not contain Fim3 [47]. A recent study demonstrated that Fim2 and Fim3 are immunogenic antigens, and that individuals recently infected with pertussis had greater anti-Fim3 IgG concentrations than anti-Fim2 IgG concentrations, consistent with the current predominance of Fim3 strains [41]. Based on this finding, there is a clear need for the improvement of currently used ACVs. Specifically, the inclusion of Fim3 (Fim3A and/or Fim3B) in ACVs may be an effective way to reduce the number of current circulating *B. pertussis* strains, including Fim3B strains. Although Fim2 has been shown to be a protective antigen, the protective immunogenicity of Fim3 is still unknown [48]. Further study of this topic will be required to evaluate the effectiveness of Fim3 as a protective antigen.

In the present study, the genetic diversity of the *B. pertussis* population decreased after the 2008−2010 pertussis epidemic. This decrease reflects the expansion of the MT27a strain type and the disappearance of MT27d strains. The MT27a strains carried the same *ptxP3*, *ptxA1*, and *prn2* alleles as the MT27d strains, but additionally carried *fim3A*. Significant changes in the *B. pertussis* population were previously observed in a pertussis epidemic in the Netherlands, and this study suggested that strain surveillance may serve as an early detector of pertussis epidemics [24]. Here, we observed significant changes within the *B. pertussis* population during the 2008−2010 epidemic, lending further support for an early warning system of future pertussis epidemics.

In conclusion, the prevalence of *B. pertussis* MT27d strains temporarily increased during the 2008−2010 pertussis epidemic in Japan. The MT27d strains carried the same virulence-associated allelic variants (*fim3B*, *ptxP3*, *ptxA1*, and *prn2*) as recent epidemic strains observed in Australia, Europe, and the US. *B. pertussis* MT27d strains, therefore, likely have the potential to cause large epidemics in other countries, and, hence, further study and strain surveillance of the MT27d strain type is warranted.

## Supporting Information

Table S1 **Characteristics of** ***Bordetella pertussis*** **study isolates.**
(XLSX)

Table S2 **PCR primers in this study.**
(XLSX)
